# Rift Valley Fever in Ruminants, Republic of Comoros, 2009

**DOI:** 10.3201/eid1707.102031

**Published:** 2011-07

**Authors:** Matthieu Roger, Sébastien Girard, Abdourahime Faharoudine, Mohamed Halifa, Michèle Bouloy, Catherine Cetre-Sossah, Eric Cardinale

**Affiliations:** Author affiliations: Centre for Scientific Research and Intelligence on Emerging Infectious Diseases in the Indian Ocean, Sainte-Clotilde, La Réunion Island, France (M. Roger, E. Cardinale);; French Agricultural Research Center for International Development (CIRAD), Montpellier, France (M. Roger, S. Girard, C. Cetre-Sossah, E. Cardinale);; CIRAD, Mamoudzou, Mayotte (S. Girard);; Vice-President, Ministry of Agriculture, Fisheries, Environment, Industry, Energy and Handicraft, Moroni, Republic of Comoros (A. Faharoudine, M. Halifa);; Institut Pasteur, Paris, France (M. Bouloy)

**Keywords:** Rift Valley fever virus, Republic of Comoros, island, ruminant, zoonoses, Indian Ocean, viruses, letter

**To the Editor:** Rift Valley fever (RVF) is caused by a *Phlebovirus* (family *Bunyaviridae*) transmitted by a wide range of mosquitoes (*1*). This zoonotic disease is present in Africa, the Middle East, and Madagascar. Infections by RVF virus (RVFV) in ruminants cause massive abortions in livestock and high death rates in young animals, which result in major economic consequences. Humans are infected by mosquito bites, contact, or inhalation of aerosols. RVF is frequently unapparent or mild for humans, inducing an influenza-like illness that occasionally leads to more serious complications such as hemorrhage, meningoencephalitis, retinopathy, or even death (*2*).

Cattle are socially important in Republic of Comoros because massive slaughtering occurs during traditional wedding ceremonies known as “Grands Mariages,” especially on the main island, Grande Comore. Because of low meat production (only 20,000 head of local cattle), a large number of live ruminants enter Grande Comore from Anjouan and Mohéli, the other 2 islands of the Republic, from Madagascar and Tanzania without quarantine or any other preliminary veterinary control.

We report results from a serosurvey of the ruminant populations on the 3 islands of the Republic of Comoros during the 2009 dry season (April–August). A total of 488 blood samples were collected from randomly selected sheep, goats, and horned cattle and sent to laboratory facilities in Mayotte to be tested by an RVFV immunoglobulin (Ig) G competitive ELISA (*3*). Fifty IgG RVFV-negative and -positive serum samples were randomly selected for confirmation by a seroneutralization assay using the reference method described in the World Organisation for Animal Health manual (*4**,**5*).

Of the 488 serum samples tested, 160 were positive for RVFV, for a seroprevalence of 32.8% (95% confidence interval [CI] 28.6%–36.9%). The 3 species were positive for IgG, with prevalence of 30.6% (95% CI 24.2%–37.1%) for cattle, 33.5% (95% CI 27.6%–39.3%) for goats, and 39.0% (95% CI 24.1%–54.0%) for sheep. Using a χ^2^ test, we found no statistically significant differences regarding species and gender, but more adults than young animals were seropositive for RVFV IgG (p<0.001). Significant differences in RVFV seroprevalence were found between islands (p<0.005), especially between Anjouan (26.6%; 95% CI 20.0%–33.3%) and Moheli (45.8%; 95% CI 31.7%–59.9%); p = 0.011). Of the 50 samples tested in seroneutralization, 31/31 (100%) of RVFV IgG ELISA-positive serum samples were confirmed as positive for RVFV (Table).

**Table:** Cross-sectional Rift Valley fever seroprevalence in sheep, goats, and horned cattle, Republic of Comoros, April–August 2009*

Animal	Positive IgG results by animal age, %	Positive IgG results by sex, %	No. animals positive by IgG/no. tested (%)				Animals with positive IgG results confirmed by SN
			Grande Comore	Anjouan	Mohéli	Total	
Sheep	Y, 25.0; A, 42.4	M, 12.5; F, 45.5	3/5 (60.0)	9/26 (34.6)	4/10 (40.0)	16/41 (39.0)	2/2
Goats	Y, 6.1; A, 43.2	M, 24.7; F, 37.4	55/139 (39.6)	20/97 (20.6)	9/15 (60.0)	84/251 (33.5)	17/17
Homed cattle	Y, 15.1; A, 38.5	M, 29.1; F, 31.2	35/127 (27.6)	16/46 (34.8)	9/23 (39.1)	60/196 (30.6)	12/12
Total	Y,11.4; A, 41.1	M, 25.7; F, 35.3	93/271 (34.3)	45/169 (26.6)	22/48 (45.8)	160/488 (32.8)	31/31

*Serum positivity was established when titers were >1:10. Ig, immunoglobulin; SN, seroneutralization; Y, young (before reproductive age); A, adult.

The serologic evidence of RVFV circulation in the ruminant population of the Republic of Comoros is in accordance with the epidemiologic situation described in other countries in the area. Actually, the serosurvey was implemented after the RVF outbreaks reported in several countries in eastern Africa in 2007 during El Niño rains (*6*). In August 2007, RVFV was detected in a young person from Comoros, and indigenous transmission of RVFV in Mayotte was confirmed in 2008 (*7*). RVF outbreaks were also reported in Madagascar during the 2008 and 2009 rainy seasons (January–May 2008 and November–March 2009) (*8*). To our knowledge, no circulation of RVFV in Republic of Comoros has been reported despite frequent legal and illegal movements of populations and goods between Republic of Comoros and eastern Africa, Mayotte, Madagascar, and the others islands of the area. With 1 of 3 ruminants having been in contact with RVFV, our results suggest that the human population in these islands have likely been widely exposed to this virus. However, several questions remain unanswered: Was RVFV recently introduced in the country? Has the virus settled down in a local reservoir for years without major clinical consequences before reemerging thanks to favorable conditions? Actually, no massive abortions in livestock or high death rate in young animals have been notified so far by the Comorian Sanitary Services. Therefore, the origin of infection is presently unknown because animals could have been infected on the island or in another country from where they have been imported.

Because live ruminants have been imported from neighboring countries for 20 years, the risk of introducing new diseases in the country is high. Despite efforts of the Comorian sanitary services, the Republic of Comoros is particularly vulnerable to pathogens intrusion. Blackleg (1970, 1995) and the contagious ecthyma (1999) were probably introduced into the country by live ruminants imported from Madagascar (*9*). Since 2002, importation of live animals from Tanzania has been common, increasing the risk of introducing continental pathogens or vectors as illustrated with outbreaks of East Coast fever in 2003 and 2004 in Grande Comore (*10*). RVFV circulation presented in this study is another example of the exposure of the Republic of Comoros to emerging pathogens and potentially bears major consequences for the local economy and for public health. The improvement of the Comorian veterinary services and the setting up of surveillance programs are essential to limit the risk of introducing devastating diseases in the area.

## References

[1] Turell MJ, Linthicum KJ, Patrican LA, Davies FG, Kairo A, Bailey CL. J Med Entomol. 2008;45:102–8.Vector competence of selected African mosquito (Diptera: Culicidae) species for Rift Valley fever virus. 10.1603/0022-2585(2008)45[102:VCOSAM]2.0.CO;2 18283949

[2] Swanepoel R, Coetzer JAW. Rift Valley fever. In: Coetzer JAW, Thomson GR, Tustin RC, editors. Infectious diseases of livestock. Oxford (UK): Oxford University Press; 2004. p. 1037–70.

[3] Paweska JT, Mortimer E, Leman PA, Swanepoel R. An inhibition enzyme-linked immunosorbent assay for the detection of antibody to Rift Valley fever virus in humans, domestic and wild ruminants. J Virol Methods. 2005;127:10–8. 10.1016/j.jviromet.2005.02.008 15893560

[4] World Organisation for Animal Health. Rift Valley fever. In: OIE manual of diagnostic tests and vaccines for terrestrial animals, vol. 1, 5th ed. Paris: Office International des Epizooties; 2004. p. 185–94.

[5] Digoutte JP, Calvo-Wilson MA, Mondo M, Traoré-Laminzana M, Adam F. Continuous cell lines and immune ascitic fluids pools in arbovirus detection. Res Virol. 1992;143:417–22. 10.1016/S0923-2516(06)80135-4 1297177

[6] Outbreaks of Rift Valley fever in Kenya, Somalia and United Republic of Tanzania, December 2006–April 2007. Wkly Epidemiol Rec. 2007;82:169–78. 17508438

[7] Sissoko D, Giry C, Gabrie P, Tarantola A, Pettinelli F, Collet L, Rift Valley fever, Mayotte, 2007–2008. Emerg Infect Dis. 2009;15:568–70. 10.3201/eid1504.081045 19331733 PMC2671425

[8] Andriamandimby SF, Randrianarivo-Solofoniaina AE, Jeanmaire EM, Ravololomanana L, Razafimanantsoa LT, Rakotojoelinandrasana T, Rift Valley fever during rainy seasons, Madagascar, 2008 and 2009. Emerg Infect Dis. 2010;16:963–70. 20507747 10.3201/eid1606.091266 PMC3086256

[9] Timmermans E, Ruppol P, Saido A, Onclin M. Influence du marché international et des stratégies d’approvisionnement en viande sur les risques d’importation de maladies. Le cas de l’ecthyma en République Fédérale Islamique des Comores. 2000 [cited 2010 Dec 1]. http://www.vsf-belgium.org/docs/ecthyma_contagieux.pdf

[10] De Deken R, Martin V, Saido A, Madder M, Brandt J, Geysen D. An outbreak of East Coast fever on the Comoros: a consequence of the import of immunised cattle from Tanzania? Vet Parasitol. 2007;143:245–53. 10.1016/j.vetpar.2006.08.018 16996692

